# Metagenomics or Metataxonomics: Best Practice Methods to Uncover the Sinus Microbiome

**DOI:** 10.1002/alr.23617

**Published:** 2025-05-30

**Authors:** Isabella Burdon, George Bouras, Kevin Fenix, Kenny Yeo, James Connell, Clare Cooksley, Emma Barry, Sarah Vreugde, Peter J. Wormald, Alkis J. Psaltis

**Affiliations:** ^1^ Department of Surgery Otolaryngology Head and Neck Surgery University of Adelaide Adelaide South Australia Australia; ^2^ Department of Otolaryngology The Basil Hetzel Institute for Translational Research Woodville South South Australia Australia; ^3^ Department of Surgery Otolaryngology Head and Neck Surgery The Queen Elizabeth Hospital, Central Adelaide Local Health Network Adelaide South Australia Australia

**Keywords:** bacteriology, chronic rhinosinusitis, sinusitis

## Abstract

- Long‐read‐metagenomic sequencing is the best method for analyzing the sinus microbiome.
- 16s‐rRNA‐sequencing (both long and short read) results in PCR amplification bias that significantly distorts the sinus microbiome.

## 1 Introduction

The sinus microbiome is a rapidly growing area of research, with at least 70 sequencing studies published to date. However, this increase in microbiome literature has coincided with greater discrepancies in results. There remains no clear consensus as to which bacteria constitute a healthy or unhealthy sinus microbiome [1]. Microbiome sequencing is complex, and these discrepancies may arise at any stage: from sample collection, DNA extraction, library preparation, sequencing platform selection to methods of data analysis. To address this issue, our department generated the largest international sinus microbiome study to date, standardizing this workflow at all stages [2, 3]. Despite these efforts, we were still unable to distinguish between healthy and unhealthy microbiomes. In a disease often clinically associated with the presence of pathogens, we realized that understanding this issue would require a closer examination of our sequencing methods. Without a publicly available sinus‐specific mock community, it has not been possible to benchmark sequencing workflows in the context of the sinus. In this research note, we present the first sinus‐specific mock community, used as a positive control to benchmark sequencing workflows. This work aims to inform sequencing method selection.

## 2 Methods

### 2.1 Generation of Mock Community

The growth curves of nine bacterial strains were characterized by serial OD600 and CFU measurements across five timepoints. To create the mock communities, the desired CFUs of each bacterium was extracted from overnight liquid cultures and combined. These were pelleted to form three biological replicates of the mock community at a total bacterial load of 2.7 × 10^9^ CFUs (mocks A–C). An aliquot of each of these was set aside to create three technical replicates at a bacterial load of 2.7 × 10^8^ CFUs (mocks D–E). Mocks D–E were then spiked with human cells (5 × 10^5^ cells). The PureLink Microbiome DNA Kit (ThermoFisher) was used for DNA extraction.

### 2.2 Sequencing Methods

The same DNA samples for mocks A–E were used for all sequencing workflows. Five sequencing methods were tested: (1) short‐read‐16s using a V3–V4 primer on the Illumina MiSeq platform at AGRF; (2) long‐read‐16s using the standard ONT‐whole‐16s primer and PCR barcoding (SQK‐16S024, ONT) on the ONT GridION; (3) long‐read‐16s using a customized KAPA‐whole‐16s primer and native barcoding on the ONT GridION; (4) short‐read metagenomic sequencing on the Illumina MiSeq platform at AGRF; and (5) long‐read metagenomic sequencing using the native barcoding kit (SQK‐NBD114.24, ONT) on the ONT GridION.

### 2.3 Data Analytics

Short‐ and long‐read shotgun samples were depleted of human reads using Minimap2 (v 2.28‐r1209) and profiled using Sourmash (v4.8.5) and the GTDB (v08‐RS214). Long‐read 16s samples (ONT and KAPA) were analyzed using emu (v3.4) and its default database. Short‐read 16s samples were profiled using QIIME2 (v2023.9) and the SILVA database (v 138.1). Data normalization was not performed; this was done to facilitate the evaluation of each method's performance at its maximum sequencing yield. Statistical analyses, including PERMANOVA, Bray–Curtis dissimilarity, and Wilcoxon rank‐sum tests, were conducted using scipy and skbio python packages and visualized with matplotlib.

## 3 Results

### 3.1 Description of the Data Set

**FIGURE 1:**
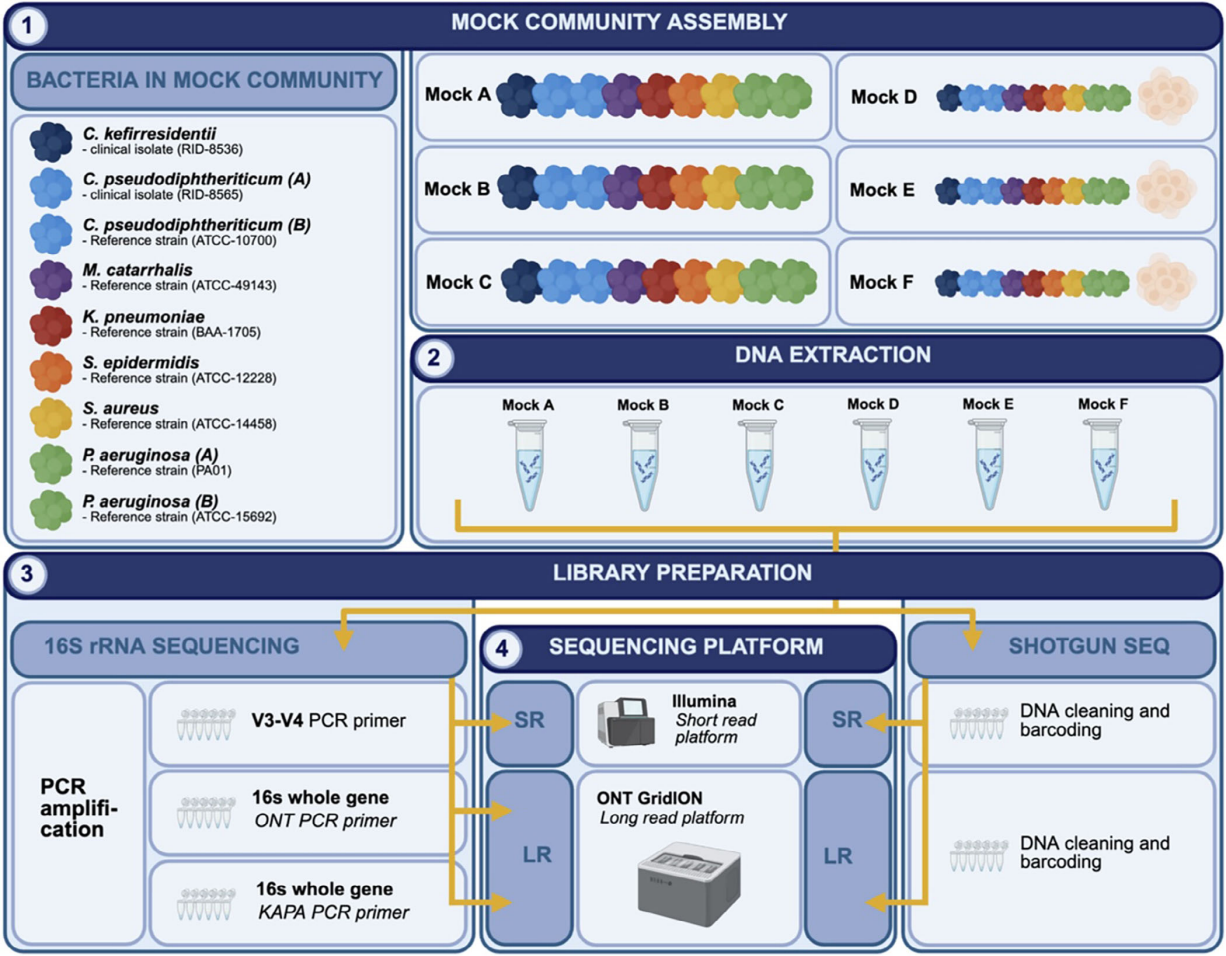
Schematic demonstrating the mock community benchmarking workflow to test five sequencing methods, and key steps are as follows: (1) mock community assembly—sinus relevant bacteria were selected for inclusion in mocks with the use of clinical isolates and type strains. Three biological replicates of the mock community were made (mocks A–C), and an aliquot of each of these was then set aside as a technical replicate and spiked with sterile bronchial cells (mocks D–F); (2) DNA extraction of mock communities; (3) methods of library preparation; (4) sequencing platform.

The dataset generated in this study represents six replicates of the sinus mock community sequenced by five different methods. The key differentiating steps for each workflow are illustrated in Figure 1.

### 3.2 Taxonomic Profiles of the Mock Community

**FIGURE 2:**
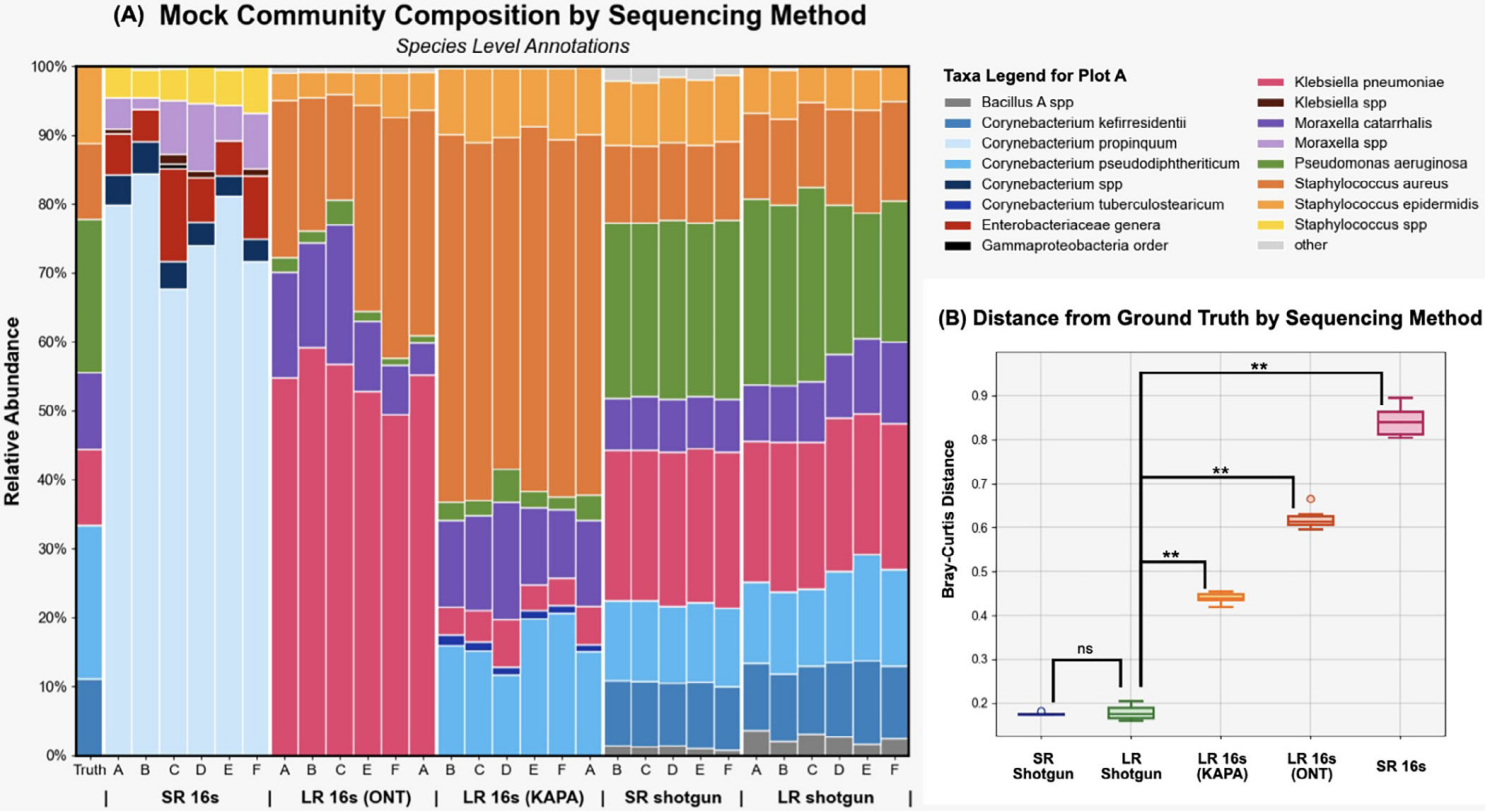
(A) Stacked bar plots showing species‐level community composition of each mock community replicate (mock A through F), grouped by sequencing method. (B) Bray–Curtis distances to the ground truth for each mock community replicate grouped by sequencing method (**p value < 0.001). PERMANOVA based on Bray–Curtis distances: pseudo‐*F* = 317.455, *p* = 0.001.

This benchmarking study finds that shotgun (metagenomic) sequencing is a significantly better approximation of the ground truth than 16s sequencing (*p* < 0.001), as illustrated by Figure 2. In this study, both long‐read and short‐read shotgun workflows identified all relevant species, and the relative abundance of sequenced taxa better reflected that of the input mock community (mean Bray–Curtis: 0.18 and 0.18).

Short‐read‐16s sequencing only resolved to the genus level and was the most dissimilar from the ground truth (mean Bray–Curtis: 0.84). This method saw a threefold overestimation of *Corynebacterium*, falsely identified *Enterobacter*, and was unable to detect *Pseudomonas*. Long‐read‐16s‐ONT sequencing saw a fivefold overestimation of *Klebsiella pneumoniae* and twofold overestimation of *Staphylococcus aureus* (mean Bray–Curtis: 0.62). Most concerningly, *Corynebacterium* species were not detected at all by this method. Long‐read‐16s‐KAPA was able to identify all key species except *Corynebacterium kefirresidentii*. Relative abundance estimates deviated from the ground‐truth in this method: the most notable differences were a fourfold overestimation of *S. aureus*, and underestimation of *K. pneumoniae and Pseudomonas aeruginosa* species (mean Bray–Curtis: 0.44).

## 4 Discussion

The results of this study suggest a change in best practice for sinus microbiome sequencing. The existing sinus microbiome literature has only used 16s rRNA sequencing methods. However, in this paper, we demonstrate that PCR amplification of the 16s rRNA gene distorts the sinus microbiome such that it is misrepresentative of the ground truth. This use of different sequencing workflows may explain the lack of consensus in the existing literature. In support of this, we point to a substantial body of literature that demonstrates PCR amplification bias in other niches of the human microbiome [4, 5, 6, 7, 8].

In each 16s (metataxonomic) workflow examined in this study, the microbiome profiles diverged both from the ground‐truth and from the profiles produced by other 16s workflows. Two 16s methods were clearly unsuitable in the sinuses; short‐read‐16s sequencing, which dramatically overestimates *Corynebacterium*, and long‐read‐16s‐ONT sequencing, which cannot detect any *Corynebacterium*. Long‐read‐16s sequencing using the KAPA primer was the most suitable of these methods and may be preferable for studies that do not necessitate accurate relative abundance. Metagenomic sequencing is a significantly closer reflection of the ground truth and thus holds strong potential for translational research. However, this method is not without challenge or fault; historically, it has been underutilized due to expense, human genome contamination, and lack of established data analytics pipelines [9].

The field of sinus microbiome research aims to generate clinically relevant information to (1) augment our understanding of disease etiology and pathophysiology and (2) improve clinical outcomes with potential diagnostic and prognostic applications. The results of this paper conclude that these research aims may be best achieved through the use of metagenomic sequencing for sinus microbiome study.

## Conflicts of Interest

Alkis J. Psaltis worked as a consultant for Medtronic, received speakers' honorarium from Storz, Sanofi, and GSK), owned shares of Chitogel, and received sponsorship of FESS course from Storz, Medtronic, GSK, Sanofi, Stryker, and ENT Technologies. The remaining authors declare no conflicts of interest.
